# Deubiquitylase OTUD3 Mediates Endoplasmic Reticulum Stress through Regulating Fortilin Stability to Restrain Dopaminergic Neurons Apoptosis

**DOI:** 10.3390/antiox12040809

**Published:** 2023-03-26

**Authors:** Ling Chen, Xuejie Huan, Fengju Jia, Zhen Zhang, Mingxia Bi, Lin Fu, Xixun Du, Xi Chen, Chunling Yan, Qian Jiao, Hong Jiang

**Affiliations:** 1 Department of Physiology, Shandong Provincial Key Laboratory of Pathogenesis and Prevention of Neurological Disorders and State Key Disciplines, Physiology, School of Basic Medicine, Qingdao University, Qingdao 266071, China; 2 College of Health and Life Science, University of Health and Rehabilitation Sciences, Qingdao 266071, China

**Keywords:** OTUD3, ER stress, IRE1α, XBP1s, Fortilin

## Abstract

OTU domain-containing protein 3 (OTUD3) knockout mice exhibited loss of nigral dopaminergic neurons and Parkinsonian symptoms. However, the underlying mechanisms are largely unknown. In this study, we observed that the inositol-requiring enzyme 1α (IRE1α)-induced endoplasmic reticulum (ER) stress was involved in this process. We found that the ER thickness and the expression of protein disulphide isomerase (PDI) were increased, and the apoptosis level was elevated in the dopaminergic neurons of OTUD3 knockout mice. These phenomena were ameliorated by ER stress inhibitor tauroursodeoxycholic acid (TUDCA) treatment. The ratio of p-IRE1α/IRE1α, and the expression of X-box binding protein 1-spliced (XBP1s) were remarkably increased after OTUD3 knockdown, which was inhibited by IRE1α inhibitor STF-083010 treatment. Moreover, OTUD3 regulated the ubiquitination level of Fortilin through binding with the OTU domain. OTUD3 knockdown resulted in a decrease in the interaction ability of IRE1α with Fortilin and finally enhanced the activity of IRE1α. Taken together, we revealed that OTUD3 knockout-induced injury of dopaminergic neurons might be caused by activating IRE1α signaling in ER stress. These findings demonstrated that OTUD3 played a critical role in dopaminergic neuron neurodegeneration, which provided new evidence for the multiple and tissue-dependent functions of OTUD3.

## 1. Introduction

Parkinson’s disease (PD) is a degenerative neurodegenerative disease that mainly affects middle-aged and elderly people [1,2]. The main cause of classical motor symptoms in PD is selective death of dopaminergic neurons in the substantia nigra (SN), leading to a decrease in dopamine release from SN-striatal projections [3,4]. Up to now, the pathogenesis of PD has not been fully elucidated. Our previous study has shown that OTU domain-containing protein 3 (OTUD3) prevents PD through stabilizing iron regulatory protein 2 (IRP2), and OTUD3 knockout mice showed dopaminergic neuronal death in the SN and Parkinsonian symptoms [5]. OTUD3 is a member of the OTU subfamily of the deubiquitinases (DUBs) family, which is highly correlated with tumorigenesis. In addition, there is mounting evidence that OTUD3 is involved in a variety of diseases other than tumors, such as ulcerative colitis [6], ribosome-related quality control [7], and innate antiviral immune [8]. However, the mechanisms of dopaminergic neuronal death in OTUD3 knockout mice remain unclear yet.

As one of the largest organelles in eukaryotic cells, the endoplasmic reticulum (ER) plays a vital role in protein synthesis and storage [9]. Cells will initiate ER stress when misfolded or unfolded proteins accumulate in the cytosol [10]. To alleviate the pressure of ER stress, cells initiate the unfolded protein response (UPR). The binding immunoglobulin protein (Bip) dissociates from the three ER stress-sensing proteins: PKR-like ER protein kinase (PERK), inositol-requiring enzyme 1α (IRE1α) and activating transcription factor 6α (ATF6α) under UPR, thereby regulating the activation of downstream pathways. Bip is an ER chaperone protein and one of the key proteins that maintain the protein homeostasis of ER [11]. It corrects the misfolding and assembly and inhibits the transport of misfolded proteins or protein subunits [12,13,14]. It also binds to misfolded proteins and unassembled complexes, initiating ER-associated degradation (ERAD), responsible for UPR regulation [15].

Fortilin, also known as translationally controlled tumor protein (TCTP), is a multifunctional protein that contains 172 amino acids and consists of β-stranded core domain, α-helical domain, and a flexible loop. Fortilin is widely distributed in the cytoplasm, nucleus [16], mitochondria [17], and has various functions, such as anti-apoptosis, pro-survival, and pro-proliferation, as well as the removal of excess Ca^2+^ from cells [16,18,19,20]. Recent studies have suggested that Fortilin participates in ER stress-induced apoptosis by binding to IRE1α [21]. A study has shown mTORC1 is involved in the regulation of the level of Fortilin, the reduction of cellular Fortilin levels upon mTORC1 inhibition [22]. Overexpression of miR-27b significantly decreased Fortilin protein and gene levels in both HSC-3 and Cal-27 cell lines [23]. However, whether there are other factors regulating Fortilin still needs further research.

Our previous research demonstrated that the iron content in the SN of OTUD3 knockout mice increased by approximately two folds [5]. The abnormal iron metabolism can trigger ER stress [24]. Over the past few decades, increasing evidence has suggested that ER stress-induced cellular damage is implicated in the pathogenesis of neurodegenerative diseases [25]. In 2007, the ER stress was first discovered in dopaminergic neurons of PD patients [26]. Studies have indicated that the incidence of ER stress is closely related to the death of dopaminergic neurons in the SN [27]. Neurotoxicity of 1-methyl-4-phenyl-pyridinium (MPP^+^) and accumulation of α-synuclein (α-syn) lead to increased expression of protein disulphide isomerase (PDI), which is a marker protein of ER stress [28]. Meanwhile, elevated levels of p-PERK, C/EBP homologous protein (CHOP), and p-IRE1α can be detected in PD cell models induced by 6-hydroxydopamine (6-OHDA), MPP^+^, and rotenone [27,29,30]. p-PERK promotes the phosphorylation of eukaryotic translation initiation factor 2 alpha (eIF2α), which significantly reduces protein synthesis in the ER. p-eIF2α selectively enhances the translation of activating transcription factor 4 (ATF4) to mitigate ER stress and restore protein synthesis [31]. Under acute ER stress, the hyperactivation of PERK can increase the expression of pro-apoptotic factor CHOP by upregulating ATF4 [32,33,34,35]. p-IRE1α can catalyze the unconventional splicing of X-box binding protein 1 (XBP1) mRNA to form XBP1-spliced (XBP1s), which stimulates the expansion of ER and the synthesis of secreted proteins [36,37,38]. This evidence suggests that ER stress is a key factor contributing to dopaminergic neuron death. However, the relationship between OTUD3 and ER stress has not been reported. Whether OTUD3 affected ER stress through novel targets, thereby affecting the survival of dopaminergic neurons, remains unclear.

In this study, we investigated the role of OTUD3 in dopaminergic neuron death and further explore its underlying mechanisms. We first reported that ER stress is present in SN dopaminergic neurons in *OTUD3*^−/−^ mice. Furthermore, we explored OTUD3-induced ER stress through IRE1α pathway, which was caused by the deubiquitination of Fortilin.

## 2. Materials and Methods

### 2.1. Plasmids and Viruses

Full-length OTUD3 WT was cloned into the pCMV-Myc vectors as indicated. Lentiviruses carrying shRNA targeting human OTUD3 (Target sequence: GGACAATAACAGAAGCGAA) were from OBIO (Shanghai, China). The plasmids Myc-Fortilin, Flag-Fortilin, Flag-OTU, Flag-UBA, Flag-UBA + Tail and Flag-Tail were purchased from OBIO Technology.

### 2.2. Cell Culture and Transfection

SH-SY5Y cells were purchased from the National Infrastructure of Cell Line Resource (Shanghai, China) and cultured in MEM/F12 medium supplemented with 15% fetal bovine serum (Gibco, Billings, MT, USA) and 1% penicillin-streptomycin at 37 °C with 5% CO_2_. SH-SY5Y cell line is the subline of SK-N-SH cell line after three clones, which possesses moderate dopamine β hydroxylase activity. This cell line is used for PD research because of its human origin, catecholaminergic neuronal properties, and ease of maintenance [39]. Human embryonic kidney cell HEK293T was purchased from the National Collection of Authenticated Cell Cultures (Shanghai, China) and cultured in DMEM-High Glucose supplemented with 10% fetal bovine serum (Gibco, Billings, MT, USA) and 1% penicillin-streptomycin at 37 °C with 5% CO_2_. Cell transfection was performed using the Lipofectamine 2000 (Invitrogen, Waltham, MA, USA) reagent according to the manufacturer’s protocol.

### 2.3. Cell Viability Assessment

Cell viability was measured using Cell Counting Kit-8 (CCK-8, K1018, APExBIO, Houston, TX, USA). Cells were seeded in 96-well plates, and at the end of treatment, add 10 μL of CCK-8 solution to each well of the plate. Incubate the plate for 2 h. Measure the absorbance at 450 nm using a multimode plate reader (PerkinElmer, VICTOR Nivo, Waltham, MA, USA).

### 2.4. Trypan Blue Assay

SH-SY5Y cells that were treated with lentivirus were washed with Hank’s Balanced Salt Solution (HBSS, 14025-092, Gibco, Billings, MT, USA), then the cells were incubated with a 0.4% trypan blue (T8154, Sigma Aldrich, St. Louis, MO, USA) mixture and stained for 3–5 min in an incubator at 37 °C. Finally, all cells were rewashed with HBSS and observed under a microscope. Dead cells were stained blue. 

### 2.5. Propidium Iodide (PI) Assay

SH-SY5Y cells which treated with lentivirus were seeded at a density of 1 × 10^5^ cells/well in 48-well plates. On the following day, PI (556547, BD Biosciences, Franklin Lakes, NJ, USA) solution was added into each well for 10 min in an incubator at 37 °C and then washed three times with HBSS solution. Thereafter, the cells were visualized by an inverted fluorescence microscope.

### 2.6. Measurement of Intracellular MDA Levels

The MDA concentration measurement was based on the protocol of the MDA assay kit (Beyotime, Shanghai, China). Briefly, cells were lysed with RIPA lysis buffer and quantified protein, then 200 μL of MDA working solution was added to 100 μL of protein homogenate, then heated the mixtures at 100 °C for 15 min. After cooling to room temperature, centrifuge at 1000 g for 10 min, pipette 200 μL of supernatant into a 96-well plate, and measure the absorbance at 532 nm with a multimode plate reader (PerkinElmer, VICTOR Nivo, Waltham, MA, USA). MDA content in cells is the concentration of MDA per unit mass.

### 2.7. Flow Cytometric Measurement of Apoptosis

The cells were washed with PBS and stained with 7-Amino-Actinomycin and PE Annexin according to the manufacturer’s protocol (Bioscience, Bellingham, WA, USA). Early apoptotic cells (PE Annexin V positive, 7-Amino-Actinomycin negative), end-stage apoptosis, and death (PE Annexin V positive, 7-Amino-Actinomycin positive) were then determined by flow V cytometry.

### 2.8. Animals and In Vivo Treatment

The OTUD3 transgenic mice model was generated by the Model Animal Research Center of Nanjing University. 6–8 months old OTUD3 transgenic mice were applied in the study. Animals were maintained in controlled temperature and humidity rooms on a 12 h light/dark cycle with free access to food and water. The *OTUD3*^−/−^ mice were injected daily with TUDCA (150 mg/kg/day) (Sigma Aldrich, St. Louis, MO, USA) by intraperitoneal injection once a day for 2 weeks. Controls received injections with an equivalent volume of vehicle (0.9% NaCl). Animal experiments were carried out according to the guidelines of the National Institutes of Health Guidelines for the Care and Use of Laboratory Animals. All protocols were approved by the Animal Ethics Committee of Qingdao University (QDU-AEC-2023026).

### 2.9. RT-PCR and Quantitative PCR

Total cell RNA was prepared using Trizol reagent (Invitrogen) following the manufacturer’s instructions. 1 μg RNA was used for cDNA synthesis in a 20 μL reaction with the reverse transcription kit (Vazyme, Nanjing, China). PCRs were performed in 20 μL reaction volumes with SYBR Green PCR master mix (Vazyme, Nanjing, China) and 0.2 μM specific primers. The primer sequences used for all qPCRs are described below. Mouse Bip: 5′-TGATGCCCAGCGACAAGC-3′ and 5′-CACCCAGGTCAAACACAAG GAT-3′; Human Bip: 5′-ACCGCTGAGGCTTATTTGGG-3′ and 5′-GCTGCCGTAGGCTC GTTGA-3′. GAPDH: 5′-GCACCGTCAAGGCTGAGAAC-3′ and 5′-TGGTGAAGACGCCAGTGGA-3′.

### 2.10. Transmission Electron Microscopy Measurement of Changes in Endoplasmic Reticulum

Transmission electron microscopy was performed using a Tecnai 10 microscope (FEI, Hillsboro, OR, USA) at the Electron Microscopy Core Facility, Qingdao University. The ER thickness was quantified by measuring the distance between two membranes of the ER lumen in the Image J photographs. Briefly, values for the distance between the two membranes of ER lumen from randomly chosen five areas of each TEM photograph were averaged. 

### 2.11. Western Blotting and Immunoprecipitation

Samples from cells and animals were lysed in RIPA lysis buffer containing a protease inhibitor and phosphatase inhibitor cocktail. The protein concentration was detected by BCA kits (Thermo Fisher Scientific, Waltham, MA, USA). Samples were separated by SDS-poly acrylamide gelelectrophoresis and transferred to a PVDF membrane. After blocked with 100 g/L non-fat milk for 2 h at room temperature. The samples were then subjected to immunoblotting with the indicated antibodies. For immunoprecipitation assays, cells were lysed with HEPES lysis buffer (20 mM HEPES, pH 7.2, 50 mM NaCl, 1 mM NaF, 0.5% Triton X-100) supplemented with a protease-inhibitor cocktail (Roche, Basel, Switzerland). Immunoprecipitations were performed using the indicated primary antibody and protein A/G agarose beads (Santa Cruz Biotechnology, Santa Cruz, CA, USA) at 4 °C. The immunocomplexes were then washed with HEPES lysis buffer five times. Both lysates and immunoprecipitates were examined using the indicated primary antibodies followed by detection with the related secondary antibody. The following primary antibodies were used at the indicated dilutions/concentrations: Mouse anti-ATF6α (1:1000, sc-166659, Santa Cruz Biotechnology, Santa Cruz, CA, USA); Rabbit anti-phospho-eIF2α (Ser51) (1:000, #3398, Cell Signaling Technologies, Danvers, MA, USA); Rabbit anti-eIF2α (1:1000, #5324, Cell Signaling Technologies, Danvers, MA, USA); Rabbit anti-phospho-PERK (Thr 982) (1:000, abs137056, Absin, Shanghai, China); Rabbit anti-PERK (1:000, 3192, Cell Signaling Technologies, Danvers, MA, USA); Rabbit anti-phospho-IRE1α (1:000, ab48187, Abcam, UK), Rabbit anti-IRE1α (1:000, #3294, Cell Signaling Technologies, Danvers, MA, USA); Rabbit anti-XBP1s (1:000, #40435, Cell Signaling Technologies, Danvers, MA, USA); Mouse anti-CHOP (1:000, #2895, Cell Signaling Technologies, Danvers, MA, USA); Rabbit anti-Fortilin monoclonal antibody (1:000, ab133568, Abcam, UK); Rabbit anti-ATF4 (1:500, #11815, Cell Signaling Technologies, Danvers, MA, USA); Mouse anti-ubiquitin (1:1000, #3936, Cell Signaling Technologies); Rabbit anti-Bip (1:000, #3177, Cell Signaling Technologies, Danvers, MA, USA). Mouse-anti-OTUD3 (1:000, MABS1819, Merck Millipore, Burlington, MA, USA); Rabbit-anti-OTUD3 (1:1000, HPA028544, Sigma Aldrich, Gillingham, UK). Anti-DDDDK-tag mAb (1:1000, #M185-3L, MBL); Anti-Myc-tag mAb (1:1000, #M192-3, MBL, Tsukuba, Japan). The secondary antibodies were used for Western blotting analysis: anti-rabbit IgG-HRP and anti-mouse IgG-HRP (Santa Cruz Biotechnology, Santa Cruz, CA, USA).

### 2.12. In Vitro Ubiquitin Conjugation Assay

Cells were treated with 20 µM of the proteasome inhibitor MG132 (Calbiochem) for 8 h before sample collection. The cells were washed with PBS, pelleted, and lysed in HEPES buffer plus 1% DTT and 1% protease inhibitor. The lysates were centrifuged to obtain cytosolic proteins and incubated with anti-Fortilin antibodies for 5 h and with protein A/G agarose beads for a further 8 h at 4 °C. The beads were then washed five times with HEPES buffer. The proteins were released from the beads by boiling them in SDS-PAGE sample buffer and analyzed by immunoblotting with an anti-ubiquitin monoclonal antibody.

### 2.13. Immunofluorescence Staining

6–8-month-old *OTUD3*^−/−^ mice with 50 mg/kg sodium pentobarbital anesthetized were perfused intracardially with 0.9% NaCl followed by 4% (*w*/*v*) paraformaldehyde solution (PFA). Brains were removed and post-fixed in PFA overnight at 4 °C, then gradually transferred to 20% (*w*/*v*), and 30% (*w*/*v*) sucrose until sectioning. Sections (20 µm) were cut on a freezing microtome (CM1905, Leica, Wetzlar, Germany). After being washed three times in PBST (0.3% Tween-20 in 0.1 M PBS), sections were blocked by 10% goat serum and then incubated overnight with primary antibody of mouse anti-TH (1:500, MAB318, Merck Millipore, Burlington, MA, USA) and rabbit anti-PDI (1:200, #3501, Cell Signaling Technologies, Danvers, MA, USA) at 4 °C overnight. Furthermore, the sections were incubated in the second antibody, Alexa Fluor 555 donkey anti-rabbit IgG (dilution used = 1:500, A31572, Invitrogen, Waltham, MA, USA), Alexa Fluor 488 donkey anti-mouse IgG (1:500, A21202, Invitrogen, Waltham, MA, USA), for 1 h at room temperature, and then added DAPI for 5 min.

### 2.14. Regents

TUDCA (100 μM) (Sigma Aldrich, St. Louis, MO, USA) was dissolved in ddH_2_O. STF-083010 (40μM) (Sigma Aldrich, St. Louis, MO, USA) was dissolved in DMSO.

### 2.15. Statistical Analysis

Data sets with only two independent groups were analyzed for statistical significance using an unpaired, two-tailed *t*-test. One-way analysis of variance (ANOVA) followed by the Student Newman-Keuls test was used for comparing the difference in more than two groups. Data are presented as mean ± SEM and analyzed by SPSS and GraphPad Prism 8.0 (GraphPad Software Inc., San Diego, CA, USA). A probability of *p* < 0.05 was considered statistically significant.

## 3. Results

### 3.1. OTUD3 Deletion Induced Neuronal Apoptosis

**Figure 1:**
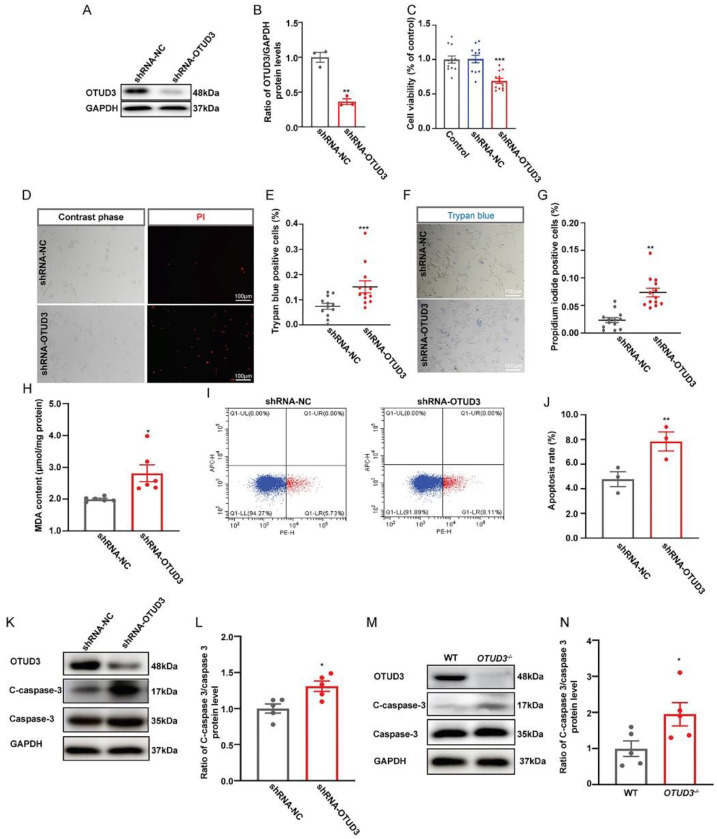
**OTUD3 knockdown induces neuronal apoptosis.** (**A**,**B**) SH-SY5Y cells with decreased OTUD3 expression were generated by lentivirally introducing shRNA-OTUD3 into the cells and characterized by western blotting, *n* = 3. (**C**) Cell viability was determined by CCK-8, *n* = 12. (**D**,**E**) PI staining and statistical analysis, *n* = 12. (**F**,**G**) Trypan blue staining and statistical analysis, *n* = 12. (**H**) The MDA content were determined by lipid peroxidation MDA assay kit, *n* = 6. (**I**) Cell apoptosis analyzed by flow cytometer with PE Annexin V/7-ADD double staining. (**J**) Statistical analysis the apoptosis rate, *n* = 3. (**K**–**N**) Western blotting and statistical analysis of the ratio of cleaved-caspase3/caspase3 in the SN of *OTUD3*^−/−^ mice and OTUD3 knockdown cells, *n* = 5. Data were mean ± SEM, *t*-test, * *p* < 0.05, ** *p* < 0.01, *** *p* < 0.001.

To observe the effect of OTUD3 on cell survival, Lv-shRNA-OTUD3 was constructed to knockdown the expression of OTUD3 in SH-SY5Y cells (Figure 1A,B). Cell viability was decreased in the OTUD3 knockdown group (Figure 1C). Meanwhile, the ratio of propidium iodide (PI) and trypan blue positive cells was significantly increased in the OTUD3 knockdown group compared with the control (Figure 1D–G). The MDA release was significantly increased in the OTUD3 knockdown group, which indicated the lipid oxidation level was up-regulated (Figure 1H). We further explored the mode of cell death induced by OTUD3 knockdown. Flow cytometry results showed that the Annexin V positive cell rate was evidently increased in OTUD3 knockdown cells (Figure 1I,J). In OTUD3 knockdown cells, the ratio of cleaved-caspase 3/caspase 3 was significantly increased, as well as in the SN of *OTUD3*^−/−^ mice (Figure 1K–N). These data indicated that knockdown of OTUD3 partially induced dopaminergic neurons deaths through apoptosis.

### 3.2. OTUD3 Knockdown Induced ER Stress

**Figure 2:**
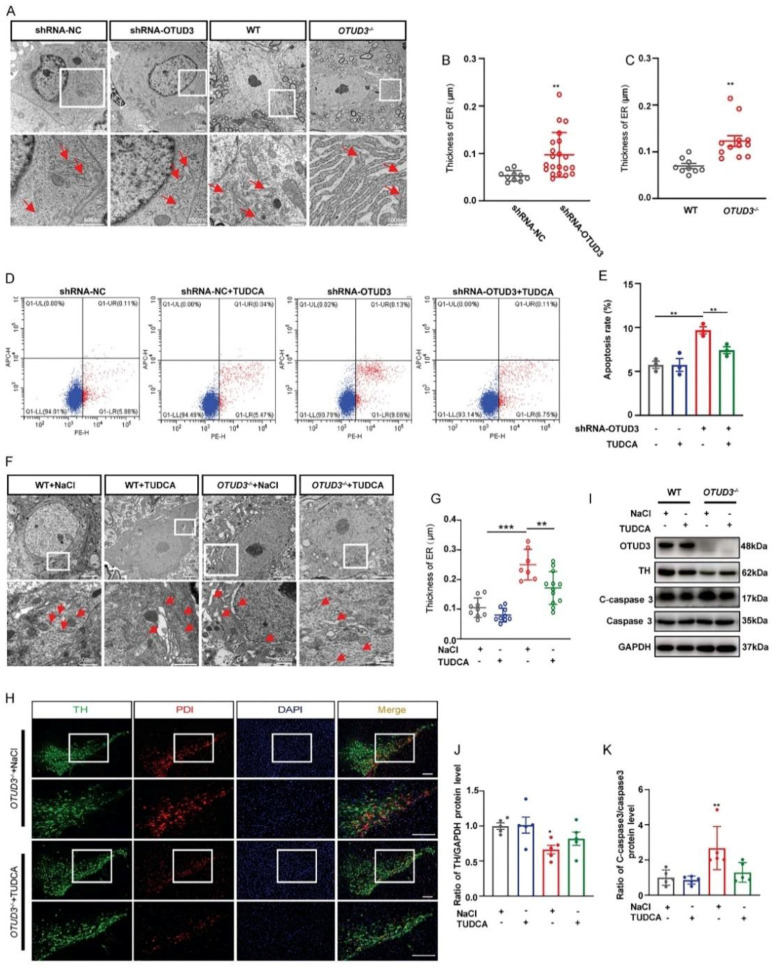
**Effects of knockdown OTUD3 on ER stress.** (**A**–**C**) Transmission electron microscope was applied to assessment of ER shape and statistical analysis of ER thickness, *n* = 9; red arrows represent the ER. (**D**,**E**) Cell apoptosis and statistical analysis of apoptosis rate by flow cytometer with PE Annexin V/7-ADD double staining, *n* = 3. (**F**,**G**) ER shape and statistical analysis of ER thickness in *OTUD3*^−/−^ mice after TUDCA treatment, *n* = 9. (**H**) Protein disulfide isomerase immunofluorescence staining of SN in *OTUD3*^−/−^ mice. Scale bar = 100μm. (**I**–**K**) Western blotting and statistical analysis of the expression of TH and the ratio of cleaved-caspase3/caspase3 proteins in *OTUD3*^−/−^ mice after TUDCA treatment, *n* = 5. Data were mean ± SEM, *t*-test, * *p* < 0.05, ** *p* < 0.01, *** *p* < 0.001.

To clarify whether OTUD3 knockdown-induced apoptosis was associated with ER stress, we observed the ER morphology and the expression of PDI (marker protein for ER stress) both in vivo and in vitro. As the main morphological manifestation of ER stress, ER expansion was significantly enlarged in OTUD3 knockdown cells. Likewise, we observed the same phenomenon in dopaminergic neurons of *OTUD3*^−/−^ mice (Figure 2A–C). Additionally, we treated OTUD3 knockdown cells with ER stress inhibitor tauroursodeoxycholic acid (TUDCA) for 24 h, the proportion of Annexin V positive cells of that was decreased (Figure 2D,E). After TUDCA treatment for two weeks, the ER lumen was markedly decreased in the dopaminergic neuron of *OTUD3*^−/−^ mice (Figure 2F,G), and the expression of PDI in dopaminergic neurons was also decreased (Figure 2H). There was an upward trend for the expression of TH protein in the SN of *OTUD3*^−/−^ mice after TUDCA treatment (Figure 2I,J), and the ratio of cleaved-caspase 3/caspase 3 showed a downward trend after TUDCA treatment (Figure 2I–K). These results suggested that OTUD3 knockdown would induce ER stress in dopaminergic neurons both in vivo and in vitro.

### 3.3. The Expression of Bip Was Not Changed In Vivo and In Vitro

**Figure 3:**
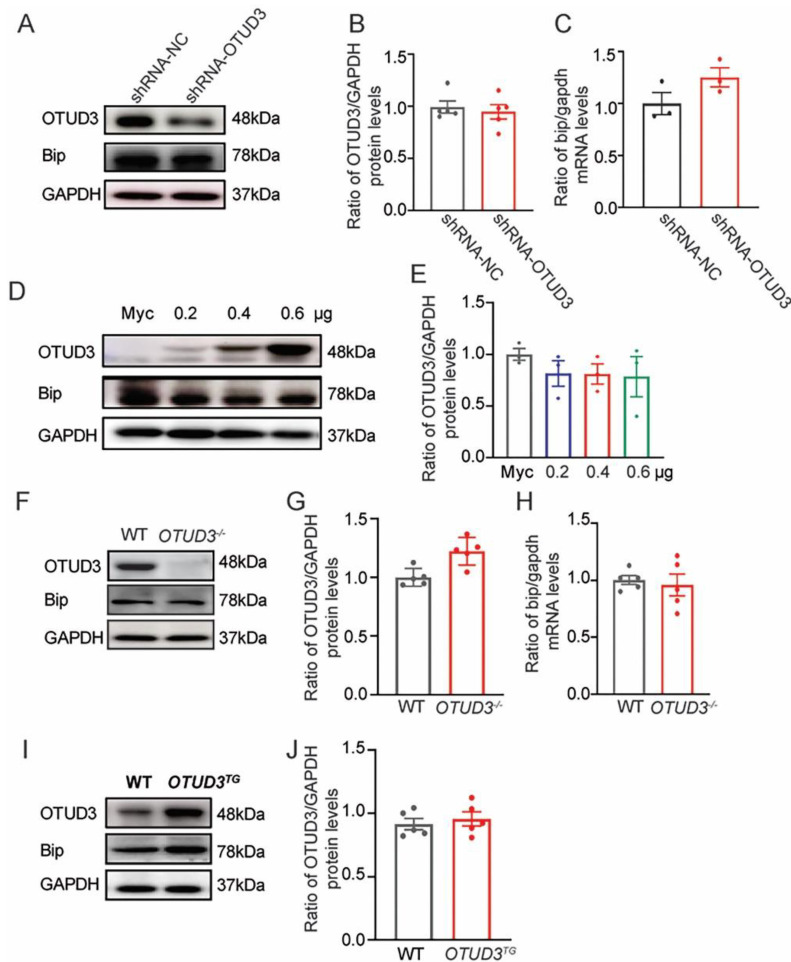
**OTUD3 knockdown-induced ER stress was independent of Bip expression change.** (**A**,**B**,**D**,**E**) Western blotting and statistical analysis of the expression of Bip in SH-SY5Y cells, *n* = 3. (**C**,**H**) The expression of bip mRNA in OTUD3 knockdown cells and the SN of *OTUD3*^−/−^ mice, *n* = 3, 5. (**F**,**G**,**I**,**J**) Western blotting and statistical analysis of the expression of Bip in the SN of mice, *n* = 5. Data were mean ± SEM.

Previous studies have shown that Bip is the target protein of OTUD3 in lung cancer cells [40]. In order to make it clear whether Bip expression changed in vivo and in vitro, we detected protein and mRNA expression levels of Bip both in OTUD3 knockdown cells and the SN of *OTUD3*^−/−^ mice. However, there were no changes in the expression levels of Bip (Figure 3A–C,F–H). Furthermore, the protein levels of Bip were also unchanged in both OTUD3 overexpression cells and the SN of OTUD3 transgenic (*OTUD3^TG^*) mice (Figure 3D,E,I,J). These data suggested that OTUD3 knockdown-induced ER stress did not affect the protein expression of Bip in the SN of mice, which might be related to the fact that the function of OTUD3 is tissue-dependent.

### 3.4. OTUD3 Knockdown Induced ER Stress via Activating IRE1α Pathway

Three classical ER stress transmembrane sensors (IRE1α, PERK and ATF6α) might be activated when ER stress occurs. To explore which ER stress pathway was activated by OTUD3 knockdown, we detected the protein expression of the three pathways separately. We found no significant changes in the expression of ATF6α-N in both OTUD3 knockdown cells and the SN of *OTUD3*^−/−^ mice (Supplementary Figure S1A−H). Although the ratio of p-PERK/PERK and the ratio of p-eIf2α/eIf2α were significantly increased in OTUD3 knockdown cells and the SN of *OTUD3*^−/−^ mice, however, the expression of PERK downstream target protein ATF4 and CHOP protein was failed to increase (Supplementary Figure S1I−P). These results suggested that the pathway of ATF6α and PERK were not activated in OTUD3 knockdown cells and the SN of *OTUD3*^−/−^ mice. 

**Figure 4:**
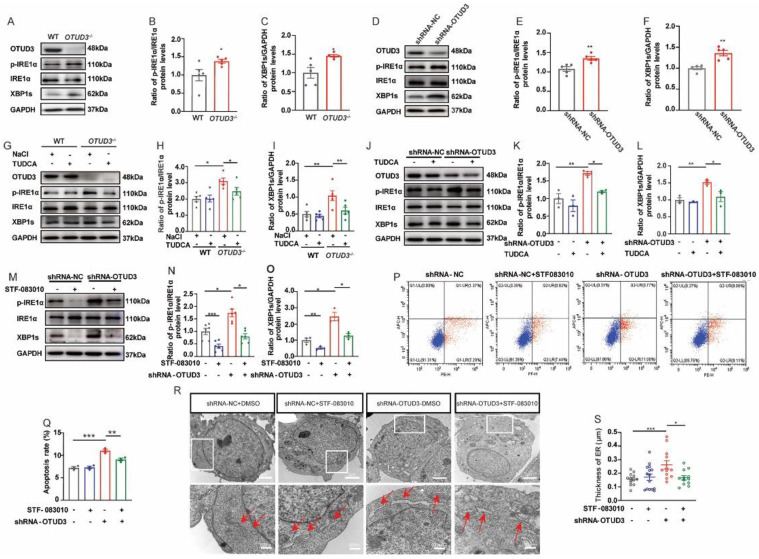
**OTUD3 knockdown-induced ER stress by actives IRE1α pathway.** (**A**–**C**) Western blotting and statistical analysis of the expression of p-IRE1α, IRE1α and XBP1s protein in *OTUD3*^−/−^ mice, *n* = 5. (**D**–**F**) Western blotting and statistical analysis of the expression of p-IRE1α, IRE1α and XBP1s protein in OTUD3 knockdown cells, *n* = 5. (**G**–**L**) Western blotting and statistical analysis of the expression of p-IRE1α/IRE1α, XBP1s in *OTUD3*^−/−^ mice (*n* = 5) and OTUD3 knockdown cells (*n* = 3) after TUDCA treatment. (**M**–**O**) Western blotting and statistical analysis of the expression of p-IRE1α/IRE1α and XBP1s in OTUD3 knockdown cells after STF-083010 treatment (*n* = 3). (**P**) Cell apoptosis analyzed by flow cytometer with PE Annexin V/7-ADD double staining. (**Q**) Statistical analysis the apoptosis rate after STF-083010 treatment, *n* = 3. (**R**,**S**) Transmission electron microscope was applied to assessment of ER shape and statistical analysis of ER lumen after STF-083010 treatment, *n* = 13, 15; red arrows represent the ER. Data were mean ± SEM, *t*−test, * *p* < 0.05, ** *p* < 0.01, *** *p* < 0.001.

Finally, we observed a significant increase in the ratio of p-IRE1α/IRE1α and the expression levels of XBP1s both in OTUD3 knockdown cells and the SN of *OTUD3*^−/−^ mice (Figure 4A–F), which were significantly reduced after TUDCA treatment (Figure 4G–L). STF-083010 blocked the increase of p-IRE1α and XBP1’s protein expression after 24 h treatment in OTUD3 knockdown cells. There was no difference between the control and the OTUD3 knockdown-STF-083010 group for the ratio of p-IRE1α/IRE1α and the expression of XBP1s (Figure 4M–O). STF-083010 treatment also inhibited the cell apoptosis of OTUD3 knockdown cells but partially reversed apoptosis after STF-083010 treatment (Figure 4P,Q). Moreover, the thickness of the ER lumen in OTUD3 knockdown cells was reduced after STF-083010 treatment, and there was no difference between OTUD3 knockdown-STF-083010 and control (Figure 4R,S). These results showed that OTUD3 knockdown-induced ER stress activated the IRE1α pathway.

### 3.5. OTUD3 Was Involved in ER Stress by Regulating the Ubiquitination Level of IRE1α Binding Protein Fortilin

**Figure 5:**
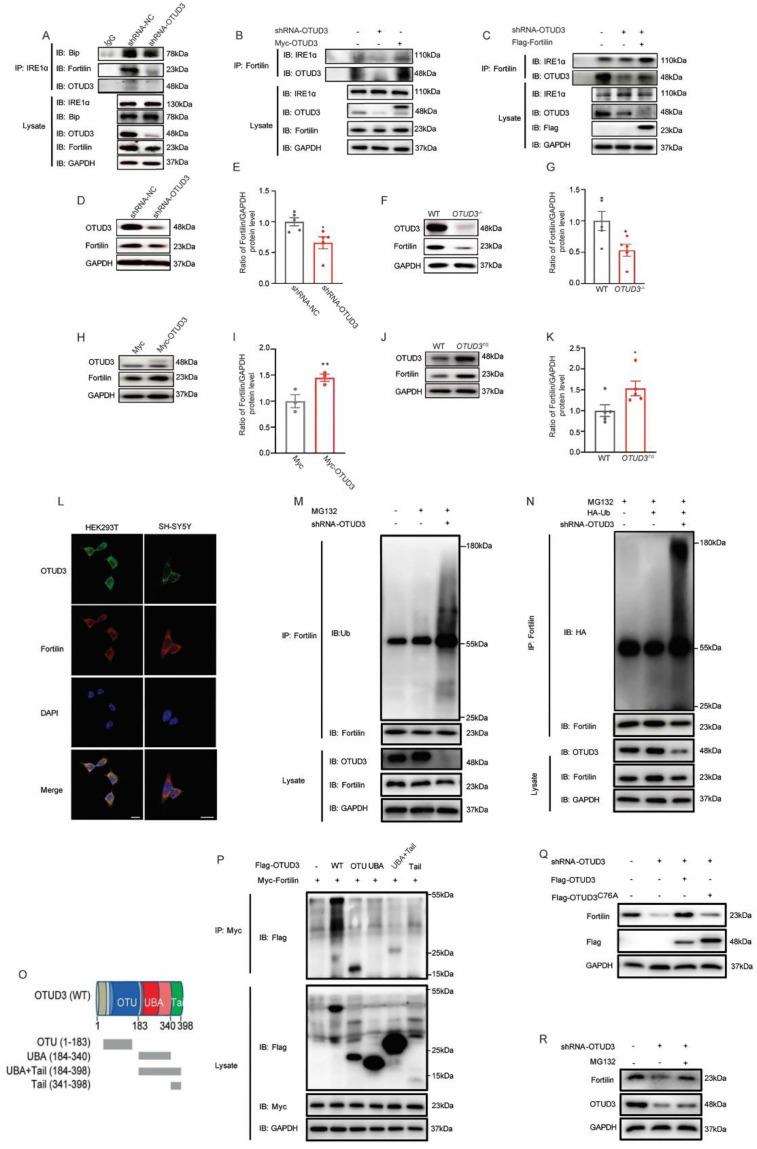
**OTUD3 regulates the ubiquitination level of Fortilin protein.** (**A**) The interaction between IRE1α and Bip, Fortilin and OTUD3 were examined immunoprecipitated with anti-IRE1α antibody. The whole-cell lysate was subjected to immunoblot with anti-IRE1α, anti-Bip, anti-OTUD3 and anti-Fortilin antibody. (**B**,**C**) The interaction between Fortilin and IRE1α and OTUD3 were examined immunoprecipitated with anti-Fortilin antibody. The whole-cell lysate was subjected to immunoblot with anti-IRE1α, anti-OTUD3 and anti-Fortilin antibody. (**D**–**K**) Western blotting and statistical analysis of the expression of Fortilin in OTUD3 knockdown cells, *OTUD3*^−/−^ mice, SH-SY5Y cells transfected with Myc-OTUD3 and OTUD3 transgenic mice, *n* = 5. (**L**) OTUD3 and Fortilin colocalized in the cytoplasm of HEK293T and SH-SY5Y cells. (**M**,**N**) The ubiquitylation levels of Fortilin was determined by in vitro ubiquitin conjugation assay. *n* = 3. (**O**,**P**) Co-IP assays were performed to map the domain of Fortilin required for interaction with OTUD3 (*n* = 3). (**Q**) Western blotting was examined the expression of Fortilin in OTUD3 knockdown cells after transfected with Flag-OTUD3^WT^ and Flag-OTUD3^C76A^. *n* = 3. (**R**) Western blotting was examined the expression of Fortilin in OTUD3 knockdown cells after treatment with MG132. *n* = 3. Data were mean ± SEM, *t*-test, * *p* < 0.05, ** *p* < 0.01.

To explore the underlying mechanism by which OTUD3 knockdown activated the IRE1α pathway, we performed co-immunoprecipitation (Co-IP) to detect protein interactions. Our findings indicated that OTUD3 did not directly interact with IRE1α, as endogenous OTUD3 was not immunoprecipitated by IRE1α antibody in shRNA-NC and OTUD3 knockdown cells (Figure 5A). We know that the activity of IRE1α is regulated by two binding proteins, Bip and Fortilin [21]. The co-precipitated signal showed that the binding ability of IRE1α to Bip was decreased in OTUD3 knockdown cells; however, the expression of Bip in whole cell lysate was not changed (Figure 5A). Meanwhile, we observed that the co-precipitated signal of IRE1α and Fortilin was decreased in OTUD3 knockdown cells (Figure 5A,B). The endogenous Fortilin could co-precipitate with OTUD3 (Figure 5B,C). To further explore the relationship between OTUD3 and Fortilin, we validated the expression of Fortilin both in OTUD3 knockdown and overexpression conditions. In OTUD3 knockdown cells and the SN of *OTUD3*^−/−^ mice, the expression of Fortilin was significantly decreased (Figure 5D–G), whereas it was increased in OTUD3 overexpression cells and *OTUD3^TG^* mice (Figure 5H–K). Moreover, co-localization studies indicated that Fortilin and OTUD3 were present in the cytoplasm of HEK293T and SH-SY5Y cells (Figure 5L). 

Ubiquitylation assays showed that OTUD3 knockdown significantly enhanced the ubiquitylation level of Fortilin in SH-SY5Y cells and HEK293T cells (Figure 5M,N). Subsequently, an exogenous Co-IP assay showed that the OTU domain of OTUD3 is sufficient to interact with Fortilin (Figure 5P). Overexpression of WT OTUD3, rather than the C76A mutant, reversed the decreased Fortilin level induced by OTUD3 knockdown (Figure 5Q). Treatment with the proteasome inhibitor MG132 also reversed the decrease of Fortilin (Figure 5R). Overall, our findings illuminated that OTUD3 did not directly bind to IRE1α, but regulated the ubiquitination level of Fortilin through its OTU domain.

### 3.6. Fortilin Alleviated OTUD3 Knockdown Induced ER Stress

**Figure 6:**
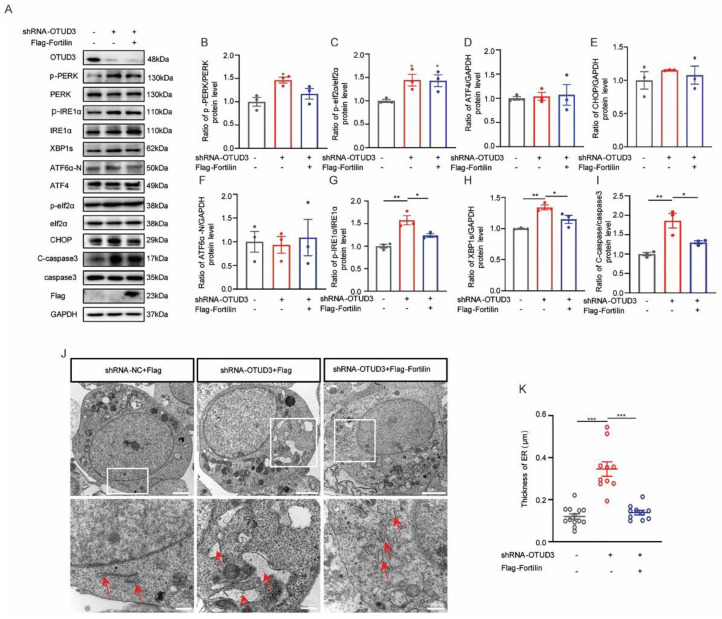
**Fortilin alleviate ER stress induced by OTUD3 knockdown.** (**A**) Western blotting to detect the ER stress-associated proteins expression. (**B**–**E**) Statistical analysis of the expression of PERK pathway-associated proteins after Fortilin overexpression in OTUD3 knockdown cells. *n* = 3. (**F**) Statistical analysis of the expression ATF6α-N proteins after Fortilin overexpression in OTUD3 knockdown cells. *n* = 3. (**G**–**I**) Statistical analysis of the ratio of p-IRE1α/IRE1α, cleaved-caspase 3/caspase 3 and the expression of XBP1s after Fortilin overexpression in OTUD3 knockdown cells. (**J**,**K**) Transmission electron microscope was applied to assessment of ER shape and statistical analysis of ER lumen after Fortilin overexpression, *n* = 10, 12; red arrows represent the ER. Data were mean ± SEM, *t*-test, * *p* < 0.05, ** *p* < 0.01, *** *p* < 0.001.

To investigate the role of Fortilin in the regulation of IRE1α activity by OTUD3, we examined the expression of the associated proteins after Fortilin overexpression. We observed that higher levels of the ratio of p-PERK/PERK and the ratio of p-eIf2α/eIf2α in OTUD3 knockdown cells were not affected by Fortilin overexpression. Notably, the ratio of p-IRE1α/IRE1α, cleaved-caspase3/caspase3, and the expression of XBP1s was increased in OTUD3 knockdown cells, and these effects were abated by Fortilin overexpression; there was no difference between the OTUD3 knockdown + Flag-Fortilin and control (Figure 6A–I). Additionally, we observed that the thickness of the ER lumen in OTUD3 knockdown cells was reduced after Fortilin overexpression (Figure 6J,K). The results indicated that Fortilin can relieve ER stress by inhibiting IRE1α pathway.

## 4. Discussion

The current work revealed that OTUD3 was involved in the regulation of ER stress by regulating IRE1α activity. OTUD3 knockdown increased the ratio of p-IRE1α/IRE1α, and the expression of XBP1s, which could be inhibited by IRE1α inhibitor STF-083010. Further investigation revealed that Fortilin was a target protein of OTUD3. Downregulation of OTUD3 reduced the binding ability of Fortilin to IRE1α, thereby activating the IRE1α pathway and inducing ER stress and neuronal apoptosis. 

OTUD3 has been identified as a tumor suppressor that is highly associated with tumorigenesis [41,42]. Our previous study found that OTUD3 knockout mice display motor deficits and nigrostriatal dopaminergic neurodegeneration, resembling the pathology of PD [5]. In the present study, we observed a significant increase in cell apoptosis after OTUD3 knockdown, consistent with our previous reports [5]. More and more evidence has shown that ER stress-induced apoptosis is an important cell death pathway for dopaminergic neurons [29,30]. We also observed abnormal expansion of ER morphology by transmission electron microscopy, both in *OTUD3*^−/−^ mice and OTUD3 knockdown cells, consistent with the previously reported ER stress-induced morphological changes [43]. At the same time, we also found that PDI, another marker protein of ER stress, was colocalized with dopaminergic neurons in *OTUD3*^−/−^ mice [44]. These results suggested that deleting OTUD3 would induce ER stress in dopaminergic neurons.

As a deubiquitylase, there are many target proteins of OTUD3, such as phosphatase and tensin homolog deleted on chromosome 10 (PTEN) [42], Bip [40], p53 [41], and actinin-4 (ACTN4) [45]. ER chaperone Bip, as one target protein of OTUD3, is a major regulator of ER stress [11]. Bip can maintain the permeability barrier of the ER during protein translocation and target misfolded proteins for retrograde translocation so that they can be degraded by the proteasome, sensing conditions of stress in the ER to activate the UPR [46]. Under physiological conditions, IRE1α, PERK, and ATF6α remain inactive by binding to Bip. Under ER stress, Bip dissociates from the three sensors, activating downstream pathways and determining cell fate [47]. In the present study, we observed that the binding ability of Bip and IRE1α was decreased after OTUD3 knockdown. However, there was no significant change in the expression of Bip in either OTUD3 knockdown or overexpression, indicating that OTUD3 did not affect the level of Bip in dopaminergic neurons. OTUD3 can stabilize Bip and promote lung tumorigenesis [40], whereas it suppresses breast tumorigenesis through stabilizing the PTEN protein [42]. This suggests that the function of OTUD3 for target proteins is distinctive in different tissues and tissue-dependent contexts. 

Under ER stress, cells activate the IRE1α, PERK, and ATF6α pathways to restore ER homeostasis or induce cell death [48,49,50]. In the present study, our results demonstrated that OTUD3 knockdown-induced ER stress and cell apoptosis through the activation of the IRE1α pathway but not the PERK or ATF6α pathways. OTUD3 knockdown increased the activation of IRE1α and the expression of XBP1s. Reversal of OTUD3 protein expression or treatment with IRE1α inhibitor STF-083010 could ameliorate IRE1α signal activation and inhibit apoptosis. The activation of IRE1α signal is closely related to apoptosis [51]. The activation of PERK and ATF6α was also related to cell apoptosis, we did not observe changes of ATF6-N protein. Although we observed an evident increase in the activation of PERK and eIf2α, the expression of downstream target proteins ATF4 and CHOP did not change. Therefore, we considered that OTUD3 knockdown could induced ER stress and cell apoptosis by activating the IRE1α pathway.

IRE1α is known to remain inactive by binding to Bip. Recent studies have shown that the IRE1α also binds to Fortilin, and when Fortilin dissociates from IRE1α, it can activate the IRE1α and lead to apoptosis [21]. As an anti-apoptotic factor, Fortilin can play anti-apoptotic function by regulating the activity of various proteins [52,53]. To verify whether the function of OTUD3 on IRE1α activity was mediated by directly regulating its ability to bind to IRE1α, Co-IP was used to detect the proteins interactions. The results showed that there was no interaction between IRE1α and OTUD3. We observed that OTUD3 knockdown could decrease the binding ability between Fortilin/Bip and IRE1α, and overexpression of OTUD3 could increase the binding ability of Fortilin and IRE1α. Additionally, we found that the expression and ubiquitination level of Fortilin protein were regulated by OTUD3, while the protein level of Bip was not influenced by OTUD3. We further found that the Fortilin was interacted with the OTU domain. These findings suggested that OTUD3 can regulate the expression of Fortilin rather than Bip, and adjust the binding ability of Fortilin to IRE1α, thus regulating IRE1α activity. In previous studies, Fortilin overexpression could inhibit the cell death and ER stress induced by thapsigargin [21]. As expected, we observed that the ER stress and cell apoptosis were alleviated by Fortilin overexpression. Our data indicated that the ER stress caused by OTUD3 knockdown might be related the Fortilin expression. Our previous research showed that the iron content is increased in the SN of *OTUD3*^−/−^ mice [5], and abnormal iron metabolism can induce ER stress [24]. These evidences suggest that OTUD3 knockout may induce ER stress by reducing the expression of IRP2 protein and up-regulating the contents of iron in neurons. Meanwhile, OTUD3 may also be involved in regulating ER stress by regulating the expression of Fortilin protein. However, there were some limitations in our present study: the ubiquitination site of Fortilin specifically regulated by OTUD3 remains to be further explored, and the changes of OTUD3 in the brain of PD patients were not clarified.

## 5. Conclusions

The present study highlights a novel role of OTUD3 in regulating ER stress. Our findings demonstrate that OTUD3 plays a crucial role in protecting cells against ER stress-induced apoptosis by regulating the level of ubiquitination of Fortilin and inhibiting the activation of IRE1α signaling. Therefore, targeting the inhibition of IRE1α signal activation could represent a promising therapeutic for PD induced by OTUD3 elimination.

## Data Availability

All data generated or analyzed during this study are included.

## Supplementary Materials

The following supporting information can be downloaded at: https://www.mdpi.com/article/10.3390/antiox12040809/s1, Figure S1: OTUD3 knockdown-induced ER stress was not depend on ATF6α and PERK pathway.
