# Supplementary material for: Deubiquitylase OTUD3 Mediates Endoplasmic Reticulum Stress through Regulating Fortilin Stability to Restrain Dopaminergic Neurons Apoptosis
Source: Antioxidants (Basel). 2023 Mar 26;12(4):809. doi: 10.3390/antiox12040809 (PMC10135230; doi:10.3390/antiox12040809)
Supplement: Supplementary file 1 [file antioxidants-12-00809-s001.zip › antioxidants-2276736-supplementary.pdf]

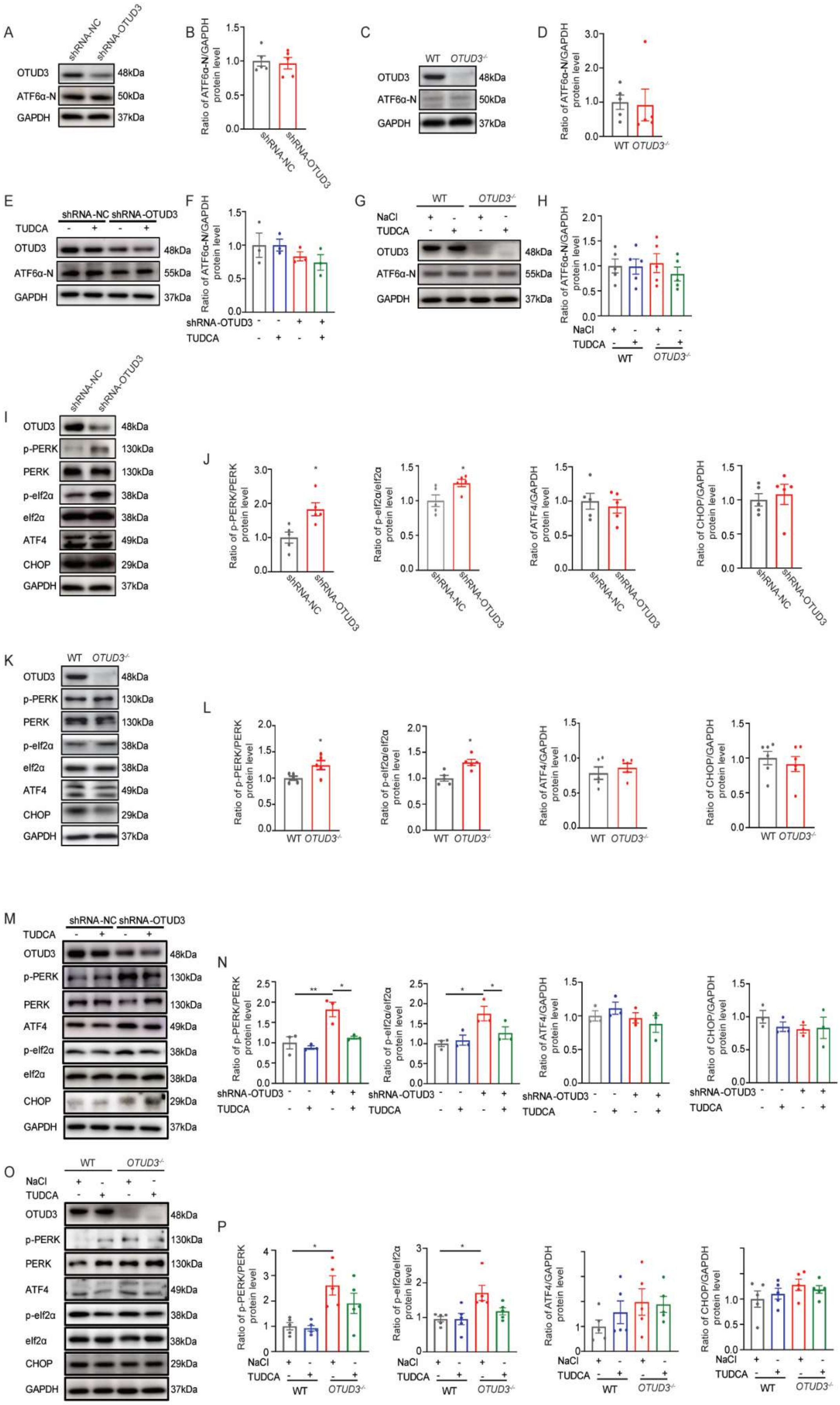
A
shRNA-NC
shRNA-OTUD3
OTUD3
ATF6α-N
GAPDH
48kDa
50kDa
37kDa
B
Ratio of ATF6α-N/GAPDH protein level
C
WT
OTUD3-/-
OTUD3
ATF6α-N
GAPDH
48kDa
50kDa
37kDa
D
Ratio of ATF6α-N/GAPDH protein level
E
shRNA-NC
shRNA-OTUD3
TUDCA
OTUD3
ATF6α-N
GAPDH
48kDa
55kDa
37kDa
F
Ratio of ATF6α-N/GAPDH protein level
shRNA-OTUD3
TUDCA
G
WT
OTUD3-/-
NaCl
TUDCA
OTUD3
ATF6α-N
GAPDH
48kDa
55kDa
37kDa
H
Ratio of ATF6α-N/GAPDH protein level
NaCl
TUDCA
WT
OTUD3-/-
I
shRNA-NC
shRNA-OTUD3
OTUD3
p-PERK
PERK
p-eIf2α
eIf2α
ATF4
CHOP
GAPDH
48kDa
130kDa
130kDa
38kDa
38kDa
49kDa
29kDa
37kDa
J
Ratio of p-PERK/PERK protein level
Ratio of p-eIf2α/eIf2α protein level
Ratio of ATF4/GAPDH protein level
Ratio of CHOP/GAPDH protein level
K
WT
OTUD3-/-
OTUD3
p-PERK
PERK
p-eIf2α
eIf2α
ATF4
CHOP
GAPDH
48kDa
130kDa
130kDa
38kDa
38kDa
49kDa
29kDa
37kDa
L
Ratio of p-PERK/PERK protein level
Ratio of p-eIf2α/eIf2α protein level
Ratio of ATF4/GAPDH protein level
Ratio of CHOP/GAPDH protein level
M
shRNA-NC
shRNA-OTUD3
TUDCA
OTUD3
p-PERK
PERK
ATF4
p-eIf2α
eIf2α
CHOP
GAPDH
48kDa
130kDa
130kDa
49kDa
38kDa
38kDa
29kDa
37kDa
N
Ratio of p-PERK/PERK protein level
Ratio of p-eIf2α/eIf2α protein level
Ratio of ATF4/GAPDH protein level
Ratio of CHOP/GAPDH protein level
shRNA-OTUD3
TUDCA
O
WT
OTUD3-/-
NaCl
TUDCA
OTUD3
p-PERK
PERK
ATF4
p-eIf2α
eIf2α
CHOP
GAPDH
48kDa
130kDa
130kDa
49kDa
38kDa
38kDa
29kDa
37kDa
P
Ratio of p-PERK/PERK protein level
Ratio of p-eIf2α/eIf2α protein level
Ratio of ATF4/GAPDH protein level
Ratio of CHOP/GAPDH protein level
NaCl
TUDCA
WT
OTUD3-/-

Figure S1. 1 OTUD3 knockdown-induced ER stress was not depend on ATF6α and PERK pathway (a-d) Western blotting and statistical analysis of the expression of ATF6α-N in OTUD3$^{-/-}$ mice and OTUD3 knockdown cells, n=5. (e-h) Immunoblotting and statistical analysis of the expression of ATF6α-N proteins in OTUD3$^{-/-}$ mice (n=5) and OTUD3 knockdown cells (n=3) after TUDCA treatment, respectively. (i-l) Immunoblotting and statistical analysis of the expression of PERK pathway associated proteins in OTUD3$^{-/-}$ mice and OTUD3 knockdown cells, n=5. (m-p) Western blotting and statistical analysis of the expression of PERK pathway associated proteins in OTUD3$^{-/-}$ mice (n=5) and OTUD3 knockdown cells (n=3) after TUDCA treatment. Data were mean±SEM, t- test, $^{*}P<0.05$, $^{**}P<0.01$.
